# Association of Sympathovagal Imbalance with Increased Inflammation and Impaired Adaptive Immunity in Bladder Cancer Patients

**DOI:** 10.3390/ijms252312765

**Published:** 2024-11-27

**Authors:** Iveta Mikolaskova, Milan Zvarik, Kinga Szaboova, Elena Tibenska, Vladimira Durmanova, Magda Suchankova, Boris Kollarik, Patrik Hesko, Patrik Palacka, Maria Bucova, Luba Hunakova

**Affiliations:** 1 Institute of Immunology, Faculty of Medicine, Comenius University in Bratislava, Odborarske namestie 14, 811 08 Bratislava, Slovakia; mikolaskova6@uniba.sk (I.M.); vladimira.durmanova@fmed.uniba.sk (V.D.); magda.suchankova@fmed.uniba.sk (M.S.); maria.bucova@fmed.uniba.sk (M.B.); 2 Department of Nuclear Physics and Biophysics, Faculty of Mathematics, Physics and Computer Science, Comenius University in Bratislava, Mlynska dolina F1, 842 48 Bratislava, Slovakia; milan.zvarik@fmph.uniba.sk; 3 Medirex, s.r.o., Galvaniho 17/C, 820 16 Bratislava, Slovakia; kinga.szaboova@medirex.sk (K.S.); elena.tibenska@medirex.sk (E.T.); 4 Department of Urology, Saint Cyril and Methodius Hospital, Antolska 11, 851 07 Bratislava, Slovakia; bkollarik@gmail.com (B.K.); patrikhesko2@gmail.com (P.H.); 5 2nd Department of Oncology, Faculty of Medicine, Comenius University in Bratislava, Kolarska 12, 812 50 Bratislava, Slovakia; patrik.palacka@fmed.uniba.sk

**Keywords:** bladder cancer, heart-rate variability, immune–inflammatory markers, autonomic nervous system, stress

## Abstract

Stress responses can impact bladder cancer (BC) outcomes via immune–inflammatory pathway modulation. This study explores heart rate variability (HRV) associations with serum immune–inflammatory biomarkers, blood count inflammatory markers, and psychosocial self-report measures in patients versus healthy controls. The TREM-1 and TREM-2 expressions on peripheral blood monocytes were analysed via flow cytometry; serum inflammatory biomarkers by ELISA; HRV (5-min ECG) pre-tumour resection; blood counts by haematology analyser; and psychosocial factors by validated questionnaires. Patients exhibited altered immune–inflammatory profiles with increased TREM-1/TREM-2, sTREM-1, sTREM-1/sTREM-2 ratio, BDNF, MCP-1, and NLR, and reduced IFN-γ, IL-10, LMR, and PMR. HRV analysis indicated sympathetic dominance (SNS, Stress indices, ACmod) and reduced parasympathetic modulation (PNS index, SDNN, RMSSD, 2UV%, DCmod, SD1). Sympathetic HRV indices correlated positively with sTREM-1, sTREM-1/sTREM-2 ratio, fractalkine, and inflammatory markers (SII, NLR, PLR) and negatively with parasympathetic HRV indices—correlations absent in controls. Only in patients, reduced physical function and social support, and higher anxiety, depression, and fatigue, associated positively with sympathetic HRV indices and inflammatory markers. This study links immune–inflammatory markers, HRV parameters, and psychosocial factors in BC, suggesting that immune and autonomic variations may relate to unfavourable outcomes. Incorporating these assessments could help tailor more personalised treatment strategies for BC patients.

## 1. Introduction

Psychoneuroimmunology research indicates that continual sympathetic and neuroendocrine system activation can lead to chronic inflammation, immune system alterations, and adverse events, hindering cancer treatment efficacy [1]. Conversely, vagal nerve activation has notable immunomodulatory effects, regulating immune responses and reducing inflammation [2].

Urothelial bladder cancer (BC), the most common urinary tract malignancy, is diagnosed as non-muscle invasive bladder cancer (NMIBC) in 75% of cases (pTa, pT1, CIS). Despite transurethral resection (TURBT) effectively treating NMIBC, recurrence rates remain high (50–70% within 5 years) [3], with a risk of progression to muscle-invasive bladder cancer (MIBC) or metastatic disease in 25% of cases [3,4]. Immediate intravesical chemotherapy or BCG immunotherapy after TURBT reduces recurrence [5,6], but managing recurrent cases requires considering individual prognostic factors, including the immune–inflammatory profile.

Peripherally, the stress response elevates immune–inflammatory cytokines, growth factors, and transcriptional regulators, influencing cancer progression [7]. Chronic stress suppresses protective immunity, induces inflammation, and enhances immunosuppressive mechanisms [7]. Increased expression of triggering receptors on myeloid cells (TREMs), particularly TREM-1 and TREM-2, has been linked to robust inflammation and poor outcomes in cancers, including renal cell carcinoma [8,9,10,11].

TREM-1 expression is linked to Th1 cell-mediated immunity and inflammation. While initially contributing to anti-tumour immunity, its prolonged presence in the tumour microenvironment (TME) is pro-inflammatory [12,13]. Its soluble form (sTREM-1) correlated with shorter survival in lung and breast cancer patients [14,15]. Conversely, TREM-2, initially considered anti-inflammatory and linked to the initiation of the Th2 cell-mediated immunity [16], is expressed in myeloid cells and promotes anti-inflammatory cytokines [10]. However, recent evidence suggests TREM-2 may suppress antitumor immunity and facilitate tumour immune evasion within the TME [10,11,17,18].

The Th1 cytokine interferon gamma (IFN-γ) is the main cytokine for maintaining Th1 immunity. Apart from ensuring anti-viral, anti-bacterial, and anti-fungal immunity, IFN-γ also enhances the functions of CD8+ cytotoxic T cells, macrophages, and natural killer (NK) cells, providing potent antitumor immunity. Moreover, IFN-γ exerts direct effects on tumours by inhibiting cell proliferation and disrupting angiogenesis within the tumour microenvironment, thereby impeding tumour growth and nutrient supply [19].

Main contra-regulatory Th1 cytokine interleukin 10 (IL-10), classified as a Th2 regulatory and anti-inflammatory cytokine, plays a great role in downregulation of Th1 antitumor immunity and inflammation [20,21]. Studies have demonstrated its suppressive role in BC immune surveillance [22].

Fractalkine, a member of the CX3C chemokine family implicated in cell migration, may have a dual role in tumorigenesis [23,24,25] and tumour immunity regulation [23,26]. In bladder tumours, elevated serum levels of CX3CL1 have been associated with increased risks of recurrence, proliferation, and invasion [27].

Brain-derived neurotrophic factor (BDNF) plays a critical role in synaptic transmission and neuronal survival in the mature central nervous system (CNS). Increased BDNF expression within tumours has been associated with poorer prognosis in various cancers [28,29]. While some studies suggest that the primary effect of BDNF is on proliferation [30], and a significant association between elevated BDNF levels and the risk of BC has been identified [31,32], some studies have also reported reduced serum BDNF levels in patients with colorectal, pancreatic and lung cancer compared to healthy controls [33,34,35].

Proinflammatory chemokine MCP-1 (monocyte chemoattractant protein-1) is also associated with tumorigenesis and tumour progression. MCP-1 facilitates invasion, metastasis, angiogenesis, and immune cell infiltration [36]. This has been demonstrated in urothelial bladder carcinoma [37,38] and various other cancer types, including breast, ovarian, hepatocellular, and prostate cancer [39,40,41,42].

Vagally-mediated HRV plays a key role in prognosticating cancer outcomes [43] by modulating immune–inflammatory pathways [2,44], mitigating sympathetic responses, and protecting across various physiological contexts [43,45,46,47]. HRV, a reflection of inter-beat interval fluctuations, represents the myocardium’s adaptability to internal and external stressors [48]. Low HRV, linked to sympathetic predominance, autonomic dysregulation [49,50] and delayed immune stress recovery, contrasts with higher HRV [51], which promotes a balanced autonomic-cardio-respiratory state and energy preservation for recovery, as seen in breast cancer patients [49,50,52,53]. Cancer treatments induce physiological and psychological stress [1], activating the sympathetic nervous system and disrupting the hypothalamic-pituitary-adrenal (HPA) axis, resulting in pro-inflammatory states that exacerbate tumour growth and trigger stress-inflammatory-cytokine responses [54], further feeding back to the CNS and impacting neuropsychological function [1].

Given these insights, this study aimed to investigate the association of selected peripheral immune–inflammatory biomarkers and inflammatory biomarkers derived from complete and differential blood counts with sympathetically and vagally-mediated HRV parameters in BC patients compared to healthy control subjects. The study also aimed to evaluate the associations between measures of self-reported health status for physical, mental, and social well-being and support, and HRV, as well as immune–inflammatory parameters, to contribute to a comprehensive understanding of the neuro-immuno-psychosocial interactions in BC patients. By exploring these relationships, this study aspires to enrich the search for novel clinical targets and facilitate the development of personalised disease management strategies.

## 2. Results

### 2.1. Basic Characteristics of Bladder Cancer Patients and Healthy Control Subjects

The study included a total of 119 participants, comprising 70 males and 49 females. Among them, the patient cohort consisted of 57 subjects (41 males, 16 females; mean age (median, IQR) = 67.77 (68.00, 64.00–73.00) years, while the control group comprised 62 subjects (29 males, 33 females; mean age = 61.40 (64.50, 52.25–72.00) years. Patients significantly differed from healthy subjects in age (*p* = 0.009) and sex (*p* = 0.009); therefore, analyses were adjusted for these confounders. The clinical characteristics of both patients and healthy subjects are outlined in Table 1.

### 2.2. Differences in TREM-1/TREM-2 Ratio on Peripheral Blood Monocytes and Serum Inflammatory Markers in Bladder Cancer Patients and Healthy Control Subjects

Bladder cancer (BC) patients had significantly higher ratios of both TREM-1%/TREM-2% (*p* < 0.001) and mean fluorescence intensity (MFI) TREM-1 MFI/TREM-2 MFI (*p* = 0.004) expressions on CD14+ monocytes compared to healthy subjects, TREM-2 MFI being significantly lower (*p* = 0.025). The serum concentrations of BDNF (*p* = 0.008) and MCP-1 (*p* = 0.025) were significantly higher in BC patients than in healthy controls, and serum concentrations of soluble TREM-1 (*p* = 0.057) and the soluble sTREM-1/sTREM-2 ratio (*p* = 0.071) showed a trend to be higher.

On the contrary, the levels of main sustaining Th1 cytokine IFN-γ (*p* = 0.001) and anti-inflammatory IL-10 (*p* = 0.009) were significantly lower in BC patients. The inflammatory marker NLR (*p* = 0.007) was also significantly higher in cancer patients, while LMR (*p* < 0.001) and PMR (*p* = 0.019) were significantly lower in patients (Table 2).

### 2.3. Differences in HRV Parameters and Psychosocial Self-Report Measures in Bladder Cancer Patients and Healthy Control Subjects

We observed higher systolic blood pressure (*p* = 0.010) and mean heart rate (beats/min) (*p* = 0.007) in BC patients compared to healthy controls (Table 1 and Table 2). BC patients exhibited significantly higher levels of HRV parameters associated with sympathetic ANS activity, including the SNS index (*p* = 0.006), Stress index (*p* = 0.027), and ACmod (ms) (*p* = 0.011).

In contrast, HRV parameters associated with parasympathetic modulation showed decreased levels in BC patients for PNS index (*p* = 0.004), SDNN (ms) (*p* = 0.014), RMSSD (ms) (*p* = 0.005), 2UV% (*p* = 0.038), DCmod (ms) (*p* = 0.006), and SD1 (ms) (*p* = 0.005). SD2 (ms) parameter (*p* = 0.029), reflecting both sympathetic and parasympathetic influences, was lower in BC patients. We also observed lower Total power (ms) (*p* = 0.002) and higher DFA-alpha 2 (*p* = 0.001) in BC patients.

Additionally, BC patients demonstrated significantly lower levels of physical function compared to healthy control subjects (*p* = 0.001) (Table 2).

### 2.4. Associations of HRV and Immune—Inflammatory Parameters

Within the patient group, HRV parameters associated with sympathetic modulation, such as SNS index, Stress index, and mean HR (beats/min) showed positive correlations with serum levels of sTREM-1 (*p* < 0.001, *p* < 0.001, *p* = 0.022), sTREM-1/s-TREM-2 ratio (*p* < 0.001, *p* < 0.001, *p* = 0.022), and fractalkine (*p* < 0.001, *p* < 0.001, *p* = 0.023), respectively.

Inflammatory parameters derived from complete and differential blood counts—SII, NLR and PLR positively correlated with linear sympathetically associated HRV indices, as follows: SII with mean HR (*p* = 0.044), ACmod (ms) (*p* = 0.033), and LF (n.u.) (*p* = 0.025), NLR with ACmod (ms) (*p* = 0.022), and LF (n.u.) (*p* = 0.015), and PLR with LF (n.u.) (*p* = 0.002).

On the contrary, SII, NLR and PLR negatively correlated with parasympathetically associated HRV parameters, as follows: SII with HF (n.u.) (*p* = 0.025) and PNS index (*p* = 0.047), NLR with RMSSD (*p* = 0.043), DCmod (ms) (*p* = 0.045), HF (n.u.) (*p* = 0.015) and SD1 (ms) (*p* = 0.044), and PLR with HF (n.u.) (*p* = 0.002).

Blood cells-to-monocyte ratios LMR, PMR and NMR positively correlated with non-linear parasympathetically associated HRV index 2UV% (*p* = 0.004, *p* = 0.012 and *p* = 0.006). LMR correlated also positively with HF (n.u.) (*p* = 0.027) and negatively with LF (n.u.) (*p* = 0.027) (Table 3).

However, these correlations were not observed in healthy controls, underscoring the role of neuroimmunoregulation in BC patients.

### 2.5. Association of HRV and Immune—Inflammatory Parameters with Clinical Characteristics

**Figure 1:**
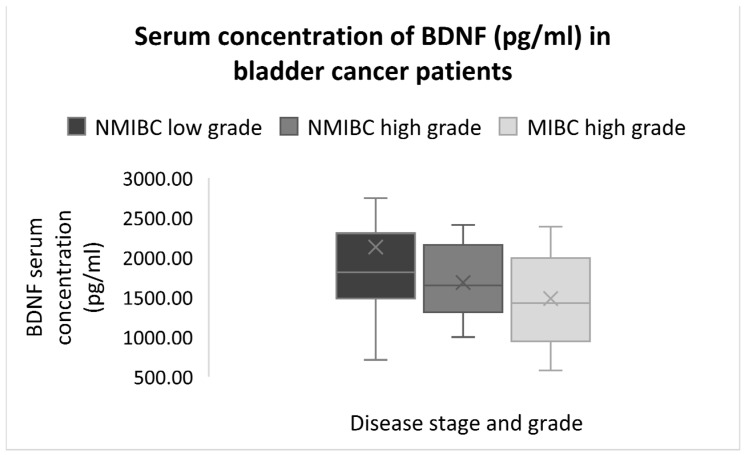
The serum concentrations of BDNF in association with disease stage and grade.

After dividing BC patients into three groups based on stage and grade: 1. non-muscle invasive bladder cancer (NMIBC) low-grade patients (pTa, carcinoma in situ/CIS, and pT1 stages), 2. NMIBC high grade (pTa, carcinoma in situ/CIS, and pT1 stages), and 3. muscle-invasive bladder cancer (MIBC) high grade (pT2 and pT3 stages), the serum levels of BDNF showed a negative association with disease stage and grade (Kendall *p* = 0.039). MIBC high-grade patients had lower levels of BDNF than NMIBC high-grade and NMIBC low-grade patients (Figure 1).

**Figure 2:**
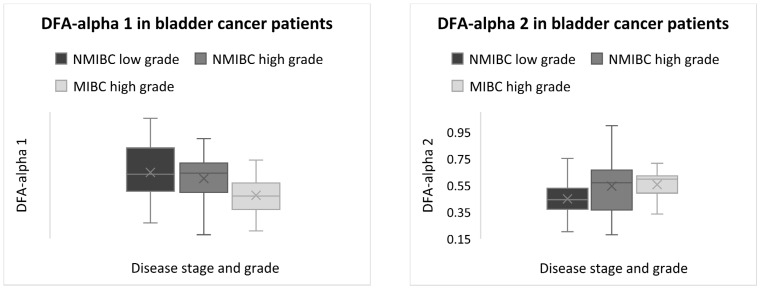
Detrended fluctuation analysis of DFA-alpha 1 and DFA-alpha 2 in association with disease stage and grade.

At the same time, reduced DFA-alpha 1, associated with an impaired ability of the heart to adapt to short-term changes by sympathetic influence, showed a negative association with disease stage and grade (Kendall *p* = 0.049), with decreased DFA-alpha 1 in MIBC high-grade patients. DFA-alpha 2, reflecting long-range correlations of fractal-like patterns in HRV over longer time scales, showed a positive association with disease stage and grade (Kendall *p* = 0.035), with increased DFA-alpha 2 in MIBC high-grade patients (Figure 2).

Although the PMR and NMR blood cells-to-monocyte ratios did not differ between NMIBC low-grade and NMIBC high-grade patients, they were significantly lower in the MIBC high-grade group (*p* = 0.059 and *p* = 0.004). Similarly, IL-10 cytokine concentration was lower in MIBC vs. NMIBC patients (*p* = 0.043).

Our data is currently not mature enough for robust survival analysis. We will revisit this analysis once a sufficient number of events have been available.

### 2.6. Association of Psychosocial Self-Report Measures with HRV and Immune—Inflammatory Parameters

In BC patients, physical function negatively correlated with the sTREM-1/sTREM-2 ratio (*p* = 0.035) and positively correlated with serum levels of BDNF (*p* = 0.049). Fatigue scores, where higher scores indicate lower fatigue, showed a negative correlation with sTREM-1 (*p* = 0.027), sTREM-1/sTREM-2 ratio (*p* = 0.024), and fractalkine (*p* = 0.030), as well as with HRV parameters, specifically the SNS index (*p* = 0.024) and Stress index (*p* = 0.037). Anxiety scores, where higher scores indicate lower anxiety, were negatively correlated with sympathetically-associated HRV parameters 0V (*p* = 0.005), mean HR (*p* = 0.003), ACmod (*p* = 0.012), LF (*p* = 0.047), LF/HF (*p* = 0.004), and SNS index (*p* = 0.010), while showing a positive correlation with vagally-mediated parameters 2UV (*p* = 0.024), SDNN (*p* = 0.043), RMSSD (*p* = 0.012), DCmod (*p* = 0.011), HF (*p* = 0.047), SD1 (*p* = 0.013), PNS index (*p* = 0.007), and sample entropy (*p* = 0.008). Similarly, depression scores, where higher scores indicate lower depression, were negatively correlated with 0V (*p* = 0.010), mean HR (*p* = 0.029), LF/HF (*p* = 0.032), and SNS index (*p* = 0.031), and positively correlated with the PNS index (*p* = 0.045) and sample entropy (*p* = 0.032), with a trend towards a positive association with 2UV (*p* = 0.054). Patients’ ability to participate in social activities negatively correlated with mean HR (*p* = 0.039). Support from friends was negatively associated with mean HR (*p* = 0.002), SNS index (*p* = 0.007), Stress index (*p* = 0.042), SII (*p* = 0.046), sTREM-1 (*p* = 0.007), sTREM-1/sTREM-2 (*p* = 0.004), and fractalkine (*p* = 0.007), and positively associated with the PNS index (*p* = 0.001) (Table 4).

These correlations were not significant in healthy controls.

## 3. Discussion

Previous studies have highlighted the implications of chronic inflammation in various malignancies, including BC [55,56], and the critical role of chronic stress in the immune dysfunction that could influence tumour behaviour has also been reported in various cancer types [57]. While recent animal research suggests that stress can significantly worsen cancer progression by influencing most hallmarks of cancer, studies in human populations and clinical trials have produced mixed results [1]. Our study brings evidence of increased sympathetic modulation together with increased inflammation and impaired adaptive immunity in bladder cancer (BC) patients. Although our current findings do not include survival data, they suggest that stress may worsen BC prognosis by negatively impacting the immune system and promoting inflammation.

In our investigation, we observed altered immune–inflammatory parameters in BC patients, characterised by an increased ratio of TREM-1 to TREM-2 (%, MFI) expressions on CD14+ monocytes, compared to healthy control subjects. TREM-1 has been linked to proinflammatory responses and facilitating tumour progression [8]. One of the mechanisms includes TREM-1 ligation initiation of a signalling cascade involving spleen tyrosine kinase (SYK), phosphatidylinositol 3-kinases (PI3K), extracellular signal-regulated kinases (ERK1/2) and mitogen-activated protein (MAP) kinases [58]. This leads to the activation of NF-κB, a key pro-inflammatory transcription factor [59], and increased production of pro-inflammatory cytokines, thus creating an inflammatory milieu with tumour-promoting effect, that further triggers signalling pathways, such as MAPK/ERK or PI3K/AKT/mTOR [58,60].

While TREM-2 has been shown to promote an immunosuppressive environment that might support tumour growth, it also facilitates tissue repair and regeneration through anti-inflammatory pathways such as SYK, NF-kB, PI3K, AKT, and MAPK [61,62]. This dual function suggests that, in certain contexts, the beneficial effects of TREM-2 in reducing inflammation and supporting tissue integrity might outweigh its potential to suppress antitumor immunity. Additionally, experimental studies have demonstrated that hematopoietic *Trem2* deletion led to lipid-associated macrophages (LAM) impairment, increased pro-inflammatory cytokine production, and exacerbated liver damage [63]. TREM-2 MFI expression was decreased in the patient cohort compared to healthy controls (*p* = 0.025).

The observed increase in the TREM-1/TREM-2 (%, MFI) ratio and decreased TREM-2 MFI expression indicates a shift toward more pro-inflammatory and tumour-promoting conditions, as demonstrated in patients versus healthy controls (*p* ≤ 0.001, *p* = 0.004). These findings are in line with investigations in other solid malignancies, such as renal cell carcinoma, hepatocellular carcinoma, and lung or colorectal cancer [64].

A shift towards a proinflammatory state in BC patients is also demonstrated by lower serum concentrations of anti-inflammatory and higher concentrations of proinflammatory cytokines in our study. Serum concentrations of IL-10 were lower in cancer patients compared to healthy subjects and were also reduced in MIBC patients. Given IL-10’s well-documented anti-inflammatory properties, its deficiency can lead to heightened inflammatory responses, potentially resulting in poor prognosis in cancer patients [65]. Reduced IL-10 levels can lead to the insufficient regulation of TNF-α and anti-tumour T cell responses, fostering tumor-promoting inflammation and contributing to cancer progression [66,67].

IFN-y, a cytokine maintaining Th1 immunity and implicated in anti-tumour responses, was found to be reduced in cancer patients [68,69,70], consistent with our own findings. This decrease in IFN-γ may impede tumour cells recognition by adaptive immunity through induction of nuclear factor-kappa B (NF-κB), signal transducer and activator of transcription 1 (STAT1) and interferon regulatory factor 1 (IRF1) transcription factors, potentially leading to impaired immune surveillance against cancer cells [19].

Our observation of elevated MCP-1 concentrations in cancer patients suggests a potential link to impaired adaptive immunity. This may involve MCP-1-driven processes, such as an increase in pro-tumour M2-like tumour-associated macrophages (TAMs) and a decrease in anti-tumour M1-like TAMs. Additionally, MCP-1 may promote TAM-dependent lymphangiogenesis and initiate angiogenesis, potentially facilitating further cancer cell invasion [36].

Our finding of significantly elevated NLR and SII in BC patients further emphasizes the presence of proinflammatory conditions in BC [56,71,72], a characteristic also observed in other types of cancers [73,74,75,76].

Blood cell-to-monocyte ratios LMR and PMR have been identified as prognostic indicators in various types of cancer. In our study, we observed lower ratios in BC patients compared to healthy controls. Previous research has shown that lower LMR has been linked to a higher risk of advanced stages in BC patients and increased 30-day mortality risk in gastric cancer patients [73,77], and lower PMR is associated with worse prognosis and poorer survival in patients with T-cell lymphoma and cervical cancer [78,79]. These blood inflammatory markers may serve as independent predictors of a higher risk of disease progression. This is in accordance with our findings of lower PMR and NMR in MIBC compared to NMIBC patients.

The HRV parameters mean HR, SNS index, Stress index, and ACmod were higher in BC cancer patients compared to healthy individuals, which may indicate a predominant sympathetic modulation and increased stress level in patients. This is consistent with our recent findings of increased stress burden in BC patients based on their electrodermal activity (EDA) and HR levels [80]. Conversely, the vagally-mediated parameters SDNN, RMSSD, DCmod, PNS index, SD1 and 2UV% were significantly decreased in patients compared to healthy controls. The SD2 parameter was also decreased in BC patients. However, this SD2 decrease likely reflects a combined influence of both sympathetic and parasympathetic nervous systems. Our analysis revealed decreased total power in BC patients compared to healthy subjects. Total power serves as a marker of the overall autonomic activity and current adaptability of an individual [81]. Beyond reflecting the balance between sympathetic and parasympathetic nervous system activity, total power provides insights into the robustness of autonomic regulation and the level of resilience to physiological and psychological stressors [82]. This aligns with the findings of others demonstrating lower total power in breast cancer patients compared to healthy subjects [83], as well as in patients with hepatocellular, lung, gastric, and oesophageal carcinoma [47].

The associations between HRV parameters and TREM in BC patients revealed positive correlations between mean HR, SNS index, Stress index, and sTREM-1 and sTREM-1/sTREM-2 ratio, suggesting sympathetic regulation of their expression. Persistent sympathetic activation, frequently observed in individuals with cancer, can lead to disrupted immune–inflammatory reactions, promoting an environment conducive to tumour growth [84]. Functionally, activation of the sympathetic nervous system (SNS) triggers the release of catecholamines (epinephrine and norepinephrine), which bind to adrenergic receptors on both immune cells and tumour cells [85,86]. The peripheral release of proinflammatory cytokines by immune cells triggers responses in afferent sensory nerves, including the vagus nerve [87]. Additionally, these cytokines transmit signals through more permeable areas of the blood-brain-barrier, facilitated by stress or pathological conditions [88]. Activated brain microglia can also recruit monocytes from the peripheral blood, influencing specific brain regions associated with mood changes and anxiety, particularly in chronically stressed individuals [87].

A non-linear parameter 2UV% showed a positive correlation with LMR, PMR, and NMR, independent prognostic biomarkers based on circulating blood cells [89]. Lower vagal nerve activity, as also indicated by reduced 2UV, may predict worse prognosis in cancers, such as breast, hepatocellular, pancreatic or lung cancer [43]. This is also supported by findings that lower LMR, PMR and NMR markers are associated with worse outcomes in cancer patients [78,90,91].

The analysis demonstrated significant associations between HRV parameters (LF, ACmod, mean HR) and inflammatory markers (NLR, SII, PLR), indicating sympathetic influence and pro-inflammatory responses. Several studies have suggested the association of LF power with sympathetic predominance activity despite existing suggestions that both parameters serve as a metric for assessing the balance between both sympathetic and parasympathetic influences. This can be attributed to different experimental setups [81]. Research has demonstrated that decreased HRV, particularly in time-domain measures, is associated with higher levels of systemic inflammation and adverse cardiovascular outcomes [92], suggesting that the interplay between autonomic regulation and inflammatory states may also be observed in cancer patients. Acceleration capacity (AC) of heart rate, which captures the shortening of the RR interval within a few successive beats [93], has been shown to have significant clinical implications. Non-survival patients with myocardial infarctions exhibited increased AC, suggesting an association of higher AC with a higher risk of mortality [94]. In our analysis, AC was positively correlated with inflammatory markers NLR and SII, further supporting the notion that elevated AC may reflect heightened inflammatory responses.

Conversely, vagally-mediated parameters, such as the high-frequency (HF) component, were negatively correlated with NLR, SII, and PLR. Moreover, the PNS index and DCmod negatively correlated with SII and NLR, respectively. Recent studies have evidenced the bidirectional association between cancer and decreased vagal nerve activity and increased sympathetic influence, resulting in heightened oxidative stress, inflammation and cancer progression [43,95]. A meta-analysis by Williams et al. showed a negative association between the HF (n.u.) band of HRV and inflammatory markers, such as TNF, IL-6, IL-1, and CRP [96]. Another study demonstrated negative correlations between HF and NLR, SII, CRP, and IL-6, giving the relation between lower vagally-mediated HRV and increased levels of inflammation [97]. Lower vagal activity, indexed by HF and higher sympathetic activity, indexed by LF, was also demonstrated in breast cancer patients [50]. Both deceleration capacity (DC) and the PNS index indicate vagal modulation. While DC focuses on short-term heart rate changes, capturing immediate autonomic responses through the analysis of instantaneous deceleration of the heart rate, the PNS index provides a broader view of sustained autonomic regulation and balance over time [98,99]. Reduced DC has been associated with increased inflammation [100] and impaired cardiac vagal modulation, resulting in an increased risk of mortality. This has been demonstrated in several diseases, including myocardial infarction, heart failure, cancer, stroke, and pneumonia [94,101,102,103].

The observed positive correlation between fractalkine and the SNS index, Stress index, and mean HR suggests a link between inflammatory processes and sympathetic dominance. Fractalkine, a chemokine involved in the recruitment and excessive activation of cytotoxic lymphocytes, plays a significant role in the pathogenesis of several inflammatory diseases by promoting the accumulation of CX3CR1-positive immune cells at sites of inflammation [26]. Increased inflammatory activity potentially induced by higher fractalkine levels could stimulate SNS activation as part of the body’s stress response. This relationship aligns with the understanding that chronic inflammation is associated with sympathetic dominance, thus contributing to stress-related physiological changes [104]. The correlation with the Stress index further underscores the impact of systemic inflammation on autonomic disruption, suggesting that fractalkine may be associated with increased sympathetic activity and stress in BC patients as well [105,106,107].

Although our findings of increased neurotrophins BDNF concentrations in BC patients compared to healthy subjects confirm the results of other studies [29,31,32,34], BDNF concentrations were decreased in high-grade MIBC patients compared to both high-grade NMIBC and low-grade NMIBC patients. Previous research indicates that the upregulation of BDNF enhances vascular endothelial cell modulation via the TrkB receptor activation of the PI3-kinase and Akt pathways. This activation mediates endothelial survival and facilitates the mobilisation and recruitment of myeloid and circulating cells [108], potentially contributing to the regeneration of disrupted tissue and cytoprotection within the tumour microenvironment of NMIBC. The frequent recurrences typical of NMIBC may necessitate ongoing tissue regeneration and repair, thus explaining the higher BDNF levels observed in NMIBC patients compared to MIBC patients, which might have a more hostile microenvironment with extensive tissue damage and less regenerative potential [109,110]. Additionally, BDNF-mediated protection is associated with the down-regulation of Bim, a pro-apoptotic protein involved in mitochondrial-mediated intrinsic apoptosis [111]. This cytoprotective mechanism may promote cell survival, proliferation and tissue integrity, further contributing to the increased BDNF levels in NMIBC patients. Supporting this, pre-operative BDNF serum levels in colorectal and pancreatic cancer patients, as well as BDNF plasma levels in patients with partial or complete resection of gliomas, were higher in earlier stages compared to advanced stages, although the differences between stages were not significant [34,112].

Additionally, there is also evidence indicating that stress can influence the expression of neurotrophic factors, particularly BDNF, in the limbic structures of the brain [113]. Chronic stress can lead to a decrease or dysfunction of BDNF, which may impair neuronal cell survival and plasticity, thereby adversely affecting hippocampal function [114]. This disruption could further modify the reactivity of the HPA axis and contribute to stress-related pathology [115]. In MIBC patients, a reduction in peripheral BDNF serum concentration suggests a potential association with increased stress and cognitive impairment, as has been shown in patients with progressed cancer [116] and more anxious coping responses to the disease [117,118]. This has also been associated with BDNF influences on GABAergic neuronal synapses, and decreased levels of this neurotrophic molecule have been associated with anxiety and depression [119].

The decreased level of BDNF within the patient cohort, particularly in those with MIBC, may be associated with the prevalence of depressive symptoms [120], although our self-report indices of depression did not show any statistical significance. In our study, higher anxiety was associated with increased sympathetic modulation and decreased vagally-mediated HRV parameters. Depression scores followed a similar pattern. It should be noted that depression can also be associated with parasympathetic predominance, particularly in clinical manifestations [121]. In our study, depression was assessed through self-report measures, which may capture anxiety-driven depressive feelings rather than clinical depression. Nevertheless, the sympathetic activation associated with increased anxiety and self-reported depression, along with the observed lower total power in BC patients, may contribute to the increased inflammatory conditions described in our study. The higher total power and lower degree of inflammation in healthy controls could explain the absence of similar correlations in this group.

Detrended fluctuation analysis (DFA) revealed lower DFA-α1 and higher DFA-α2 in MIBC high-grade compared to NMIBC high-grade and NMIBC low-grade patients. This finding indicates that conditions that enhance sympathetic activity can modify the inter-beat interval, resulting in a disruption of the fractal organisation of HRV and cardiac autonomic regulation and a decrease in α1 [122,123]. Reduced fractal complexity may result in decreased adaptability to cope with various stressors [124]. A study by Mandarano et al. showed that individuals with depressive symptoms demonstrated increased randomness in HRV, particularly reflected by elevated DFA-alpha 2 values [125]. Recent research suggests that depression may arise from disrupted cardiac vagal and sympathovagal dynamics, impacting the interplay between the central nervous system and the heart. This also highlights the complex relationship between autonomic regulation, mood disorders, and central nervous system functioning [126].

Fatigue, anxiety, and depression can create a vicious cycle where each factor exacerbates the others. For instance, fatigue can lead to inactivity, which further worsens fatigue and contributes to anxiety and depression. Our data demonstrate that fatigue scores are inversely related to sTREM-1, sTREM-1/sTREM-2, and fractalkine, as well as to sympathetically associated HRV parameters such as the SNS index and Stress index. These findings indicate that higher fatigue is associated with increased inflammatory markers and sympathetic modulation. This underscores the impact of systemic inflammation [127] and autonomic dysregulation [128] on perceived fatigue in cancer patients, as also demonstrated in breast cancer survivors [128,129,130].

Additionally, the ability to participate in social activities, which was negatively correlated with mean HR, suggests that better social engagement is associated with lower resting heart rates, reflecting a healthier autonomic state. This finding aligns with studies on the health benefits of social support and engagement [131,132,133]. Support from friends, negatively associated with sympathetic-associated HRV parameters (SNS index, Stress index, mean HR) and immune–inflammatory biomarkers (sTREM-1, sTREM-1/sTREM-2, Fractalkine, SII), and positively associated with the PNS index, underscores the protective role of social support against stress and inflammation. These associations highlight the potential therapeutic value of fostering strong social networks to mitigate stress and inflammatory responses in cancer patients [134,135,136].

Physical activity is a key factor that can break the vicious cycle of fatigue, anxiety, and depression. Regular exercise can reduce fatigue, improve mood, and alleviate anxiety and depression, leading to increased energy levels and motivation. This creates a positive feedback loop that promotes further physical activity [137]. In our investigation, the negative correlation between physical function and the sTREM-1/sTREM-2 ratio, along with a positive correlation with BDNF, suggests that impaired physical function is linked to increased pro-inflammatory conditions and enhanced neurotrophic activity. This finding aligns with previous studies showing that upregulated BDNF can enhance vascular endothelial cell modulation and facilitate the regeneration and repair of inflammation-induced disrupted tissue [108,109,110]. The interplay between physical activity, fatigue, anxiety, and depression in cancer patients is complex. Our research indicates that regular physical activity can play a significant role in improving sympathovagal balance and the inflammatory milieu, thereby managing these symptoms and enhancing overall quality of life.

Despite the insights provided by this study, several limitations warrant consideration. The sample size was relatively small, limiting the generalisation of the findings. As this study was observational, it cannot establish definitive causation. Future studies with larger cohorts are needed to validate our findings, explore additional immune–inflammatory markers, and clarify the specific role of bladder cancer in autonomic nervous system dysregulation. Moreover, longitudinal studies are warranted to elucidate the dynamic changes in immune–inflammatory profiles and HRV parameters over time and their impact on cancer progression and intervention outcomes.

## 4. Materials and Methods

The study group included 57 patients older than 18 years, originating from the Urology Clinic of St. Cyril and Methodius Hospital in Bratislava. Patients with a primary diagnosis of bladder cancer with relapse of tumour and undergoing a transurethral resection for a bladder tumour (TURBT) were analysed. In our cohort, patients with histologically proven cancer stages pTa, CIS, pT1, as non-muscle invasive bladder cancer (NMIBC), and pT2 and pT3, as muscle-invasive bladder cancer (MIBC), were enrolled. The diagnosis was approved by the hospital’s histopathologists according to the WHO 2016 classification criteria [138]. Exclusion criteria involved subjects diagnosed with diabetes mellitus, heart diseases such as coronary artery disease, left ventricular hypertrophy, valvular heart disease, cardiac pacemaker implant and other malignancies diagnosed in the previous 5 years. Subjects taking cardiac glycosides, anti-arrhythmic drugs, selective serotonin reuptake inhibitors or atropine were excluded, as well as participants with poor ECG quality or ectopic beats of more than 10 percent. Blood samples and ECG recordings for HRV analysis were obtained from the patients in the sitting position before surgical treatment one day before or on the day of surgery.

The reference cohort in our study comprised 62 unrelated volunteers. All control subjects were without any personal history of cancer, and they were randomly recruited from a larger population sample. The same exclusion criteria applied to patients were also applied to healthy control subjects. All patients and controls were Caucasians of Slovak descent. The investigations were obtained between November 2021 and November 2023 and were carried out in accordance with the International Ethical Guidelines and the Declaration of Helsinki. Written informed consent for enrolling in the study and for personal data management was obtained from all examined subjects. The study was approved by the Ethics Committees of both the Hospital of St. Cyril and Methodius and the Faculty of Medicine, Comenius University in Bratislava (125/2021).

The percentage of CD14+ TREM-1+ and CD14+ TREM-2+ monocytes (Mo) were measured by flow cytometry (Navios-Ex, Beckman Coulter, Indianapolis, IN, USA). Both the percentage and MFI (mean fluorescence intensity) of TREM expressions were analysed by Kaluza analysis software, version 1.1 (Beckman Coulter, Indianapolis, IN, USA) (monoclonal antibodies used: CD14-PC7, TREM-1-PE, TREM-2-APC/FITC and isotype controls; from Beckman Coulter, Indianapolis, IN, USA). All TREM-1 and TREM-2 analyses were performed in compliance with the flow cytometry protocol recommended by the manufacturer.

In addition to these cellular parameters, serum concentrations of various biomarkers were analysed using a sandwich ELISA test. These biomarkers included soluble TREM-1 (sTREM-1), soluble TREM-2 (sTREM-2), fractalkine/CX3CL1, brain-derived neurotrophic factor (BDNF), interferon gamma (IFN-γ), interleukin 10 (IL-10), and monocyte chemoattractant protein 1 (MCP-1). The assays were conducted in accordance with the instructions provided by the respective human Elisa kits: sTREM-1, sTREM-2, fractalkine, BDNF, IFN-γ, IL-10, and MCP-1 (all from Fine Biotech Co., Ltd., Wuhan, China).

For enhanced characterisation of inflammation and immunity, we assessed the ratios of TREM-1% to TREM-2% and TREM-1 MFI to TREM-2 MFI, as well as the ratio of serum concentration of their soluble forms sTREM-1/sTREM-2.

From complete and differential blood counts, the absolute counts of monocytes, lymphocytes, and neutrophils necessary for calculating the inflammatory biomarkers as the ratios of lymphocytes-to-monocytes (LMR), platelets-to-monocytes (PMR), and neutrophils-to-monocytes (NMR), as well as systemic immune-inflammation index (SII; neutrophils x platelets/lymphocytes), neutrophil-to-lymphocyte ratio (NLR) and platelet-to-lymphocyte ratio (PLR), were obtained using an automated haematology analyser (Mindray BC-6200, from Mindray Bio-Medical Electronics Co., Ltd., Shenzhen, China). The evaluation of these calculated parameters is encompassed, considering the established associations between inflammation, compromised immune function, and disease progression across diverse conditions, including cancer [77,78,90,139].

The ECG measurements for HRV determination were conducted in patients and healthy controls for 6 min. Participants rested in a supine position for 10 min before sitting down and starting the recording. This was achieved using the ECG device Bittium Faros 180 (Bittium Biosignals Ltd., Kuopio, Finland), with two electrodes positioned at the right first and left fifth intercostal spaces at the midclavicular line. Prior to electrode placement, the skin area was prepared with ethanol. A sampling rate of 1000 Hz was utilised to capture the electrical signal. HRV analysis was performed using the Kubios Premium system, version 3.5.0 (Kubios Oy, Kuopio, Finland) to assess the balance and the activity of the ANS using selected HRV parameters known to be associated with the sympathetic and parasympathetic branches of ANS. Indices associated with the sympathetic nervous system activity included time-domain measures of mean heart rate (HR, beats/min), modified acceleration capacity of the heart rate (ACmod, ms), frequency–domain parameter of the normalised low-frequency band (LF, n.u.), as well as non-linear parameters, the sympathetic nervous system (SNS) index, and Stress index. Vagally-mediated HRV parameters included time–domain parameters of the standard deviation of normal-to-normal interbeat intervals (SDNN, ms), root mean square of successive differences between normal heartbeats (RMSSD, ms), modified deceleration capacity of the heart rate (DCmod, ms), frequency-domain parameters of the normalised high-frequency band (HF, n.u.), and non-linear parameters, such as 2UV%—a non-linear symbolic index of transformed a time series (RR intervals) into patterns with two unlike variations, the parasympathetic nervous system (PNS) index, SD1—the standard deviation of Poincaré plot perpendicular to the line-of-identity quantifying short-term heart rate variability and SD2—the standard deviation of the Poincaré plot along the line-of-identity quantifying long-term heart rate variability and reflecting both sympathetic and parasympathetic influences. Other analysed parameters were Total power, the sum of the energy in the LF, HF and very low frequency (VLF) bands for short-term recordings, reflecting overall autonomic activity and current person adaptability. DFA-alpha1, an entropy parameter of detrended fluctuation analysis, measuring short-term correlations within the signal, and DFA-alpha 2, an entropy parameter of detrended fluctuation analysis, measuring long-term correlations within the signal.

Psychological assessment was conducted using the PROMIS–29 Profile v2.1 (Patient-Reported Outcomes Measurement Information System—29 Profile v2.1) [140] to measure patient-reported health status across physical, mental, and social well-being domains. This instrument evaluates seven domains: physical function, anxiety, depression, fatigue, sleep disturbance, social role participation, and pain interference. Symptom severity or frequency was measured on a 5-point Likert scale, ranging from 1 to 5. The questions regarding physical function and social role participation were not time-specific, while those related to the other five domains referenced the past seven days. Domain scores were calculated by summing the item scores within each domain, resulting in a possible range of 4 to 20. These scores were then converted to T-scores according to the PROMIS Adult Profile Instruments Scoring Manual [141]. Higher scores indicate better physical function and social role participation, as well as lower pain interference and less severe anxiety, depression, fatigue, and sleep disturbance.

The Multidimensional Scale of Perceived Social Support (MSPSS) [142] was employed to assess participants’ perceived social support. The MSPSS is a 12-item questionnaire evaluating support from three sources: family, friends, and a significant other. Responses are recorded using a 7-point Likert scale, ranging from 0 (strongly disagree) to 7 (strongly agree). The total score for perceived social support is calculated as the mean score of all 12 items. Subscale scores for family, friends, and significant other support are determined by summing the scores of specific items related to each subscale and then dividing by 4. Higher scores on the MSPSS indicate greater perceived social support.

### Statistical Analysis

Descriptive statistical data were expressed as the median and interquartile range (IQR). Fisher’s exact test was performed to evaluate the significance of differences between the proportions of categorical variables in the control and patient groups. Differences in the mean values of the parameters between the groups were analysed using the *t*-test or, for non-normally distributed data, the Mann-Whitney U test. Normality was assessed using the Shapiro-Wilk test in conjunction with visual inspection of quantile-quantile plots. When a parameter was dependent on age or sex, ANCOVA was used to control for these variables. If ANCOVA assumptions were violated, data were subjected to logarithmic transformation. Should the assumptions remain unmet post-transformation, the non-parametric ANCOVA alternative was implemented using the R package version 0.6-1 “fANCOVA” [143]. Partial correlation was conducted to investigate relationships between two variables while controlling for sex and age covariates using the R package version 1.1 “ppcor” [144]. Pearson’s correlation coefficient was applied for continuous variables, and Kendall’s τ coefficient was used for ordinal variables in the correlation analyses. All analyses were processed using the R environment for statistical computing [145], and a value of *p* < 0.05 was considered statistically significant.

## 5. Conclusions

In conclusion, our study highlights the associations between immune–inflammatory markers, HRV parameters and psychosocial factors, reflecting changes in sympathovagal balance in BC patients. Compromised immune function, sympathetic modulation, and psychosocial factors may impact clinical outcomes in these patients. In clinical assessment, it is necessary to consider the patient’s overall autonomic health, medical history, and psychosocial background. Our findings may provoke the design of personalised intervention strategies for stress management and improvement of impaired autonomic and immune regulation. Further research is needed to validate these findings and assess their clinical applicability in BC management.

## Figures and Tables

**Table 1:** Descriptive statistics in bladder cancer patients and healthy control subjects.

Total Number of Subjects: *n* = 119	Bladder Cancer Patients	Healthy Controls	*p* Value
	*n* = 57	*n* = 62	
Age (years—median, IQR)	68.00 (64.00–73.00)	64.50 (52.25–72.00)	0.009 *
Males (*n*)	41	29	0.009 *
Females (*n*)	16	33	
BMI (median, IQR)	28.30 (24.80–30.90)	26.50 (24.63–28.58)	0.084
Systolic blood pressure (median, IQR)	134.50 (125.00–148.25)	125.00 (115.00–135.00)	0.010 *
Diastolic blood pressure (median, IQR)	84.00 (75.75–89.25)	80.00 (75.00–88.50)	0.273
NMIBC (*n*)	48		
Low grade (*n*)	26		
High grade (*n*)	22		
MIBC (*n*)	9		
High grade (*n*)	9		

Note: BMI = body mass index; NMIBC = non-muscle invasive bladder cancer (pTa, carcinoma in situ/CIS, and pT1 stages); MIBC = muscle-invasive bladder cancer (pT2 and pT3 stages); * *p* ≤ 0.05 is considered significant.

**Table 2:** Comparison of the study variables between bladder cancer patients and healthy control subjects, adjusted for age and sex.

Total Number of Subjects: *n* = 119	Bladder Cancer Patients *n* = 57	Healthy Controls *n* = 62	*p* Value
TREM-1 MFI (median, IQR)	2.01 (1.54–2.33)	1.45 (1.19–4.10)	0.390
TREM-2 MFI (median, IQR)	0.36 (0.35–0.38)	0.41 (0.36–0.79)	0.025 *
TREM-1%/TREM-2% (median, IQR)	477.50 (174.00–831.00)	133.00 (36.89–340.19)	<0.001 *
TREM-1 MFI/TREM-2 MFI (median, IQR)	5.20 (3.41–6.47)	3.31 (2.25–4.59)	0.004 *
sTREM-1 (pg/mL) (median, IQR)	422.40 (328.60–540.80)	352.80 (252.60–435.60)	0.057
sTREM-2 (pg/mL) (median, IQR)	2176.00 (1982.35–2387.50)	2210.00 (1801.40–2682.00)	0.862
sTREM-1/sTREM-2 (median, IQR)	0.19 (0.15–0.24)	0.14 (0.11–0.19)	0.071
Fractalkine (pg/mL) (median, IQR)	1666.40 (1380.20–2548.00)	1456.00 (1337.00–1799.80)	0.203
BDNF (pg/mL) (median, IQR)	1755.40 (1410.00–2160.00)	1307.20 (1044.40–1694.40)	0.008 *
IFN-y (pg/mL) (median, IQR)	22.73 (11.50–54.34)	55.98 (48.3–103.32)	0.001 *
IL-10 (pg/mL) (median, IQR)	8.09 (4.89–76.96)	50.46 (27.30–109.58)	0.009 *
MCP-1 (pg/mL) (median, IQR)	344.40 (281.6–406.6)	290.40 (234.4–336.2)	0.025 *
NLR (median, IQR)	2.52 (1.73–3.37)	1.96 (1.5–2.48)	0.007 *
SII (median, IQR)	556.18 (406.60–897.25)	481.24 (380.53–626.19)	0.082
LMR (median, IQR)	4.00 (3.12–5.16)	5.28 (4.03–6.17)	<0.001 *
PMR (median, IQR)	565.85 (410.45–817.50)	670.59 (540.00–886.21)	0.019 *
SDNN (ms) (median, IQR)	13.67 (9.65–20.98)	20.59 (13.69–25.58)	0.014 *
Mean HR (beats/min) (median, IQR)	71.41 (65.13–78.63)	70.08 (63.51–75.23)	0.007 *
RMSSD (ms) (median, IQR)	12.02 (7.64–19.82)	17.96 (11.12–24.22)	0.005 *
ACmod (ms) (median, IQR)	−12.84 (−22.29–−8.12)	−18.39 (−28.83–−11.39)	0.011 *
DCmod (ms) (median, IQR)	12.55 (8.03–20.26)	18.13 (11.68–26.76)	0.006 *
LF (n.u.) (median, IQR)	71.00 (51.75–78.21)	65.95 (53.43–79.46)	0.875
HF (n.u.) (median, IQR)	28.79 (21.79–48.23)	34.00 (20.52–46.55)	0.875
Total power (ms) (median, IQR)	172.76 (77.95–394.8)	371.99 (178.00–588.03)	0.002 *
SD1 (ms) (median, IQR)	8.51 (5.41–14.03)	12.72 (7.87–17.15)	0.005 *
SD2 (ms) (median, IQR)	17.27 (12.29–24.88)	25.84 (17.35–31.45)	0.029 *
DFA-alpha 1 (median, IQR)	1.15 (0.95–1.40)	1.16 (0.91–1.31)	0.132
DFA-alpha 2 (median, IQR)	0.50 (0.38–0.62)	0.40 (0.29–0.49)	0.001 *
PNS index (median, IQR)	−1.21 (−1.66–−0.68)	−0.85 (−1.28–−0.4)	0.004 *
SNS index (median, IQR)	2.36 (1.41–3.61)	1.58 (0.89–2.45)	0.006 *
Stress index (median, IQR)	22.84 (16.56–28.73)	17.24 (14.56–22.51)	0.027 *
2UV% (median, IQR)	12.39 (8.57–18.41)	14.56 (9.62–22.09)	0.038 *
Physical function T score (median, IQR)	48.30 (45.50–57.00)	57.00 (48.30–57.00)	0.001 *

Note: TREM-1,2% = triggering receptor expressed on myeloid cells 1 and 2 percentage; TREM-1,2 MFI = triggering receptor expressed on myeloid cells 1 and 2 mean fluorescence intensity; sTREM-1,2 = soluble TREM; Fractalkine = Fractalkine/CX3CL1; BDNF = brain-derived neurotrophic factor; IFN-y = interferon gamma; IL-10 = interleukin 10; MCP-1 = monocyte chemoattractant protein-1; NLR = neutrophil-to-lymphocyte ratio; SII = systemic immune-inflammation index; LMR = lymphocyte-to-monocyte ratio; PMR = platelet-to-monocyte ratio; SDNN = standard deviation of normal-to-normal interbeat intervals; Mean HR = mean heart rate; RMSSD = root mean square of successive differences between normal heartbeats; ACmod = modified acceleration capacity computed as a two-point difference, DCmod = modified deceleration capacity computed as a two-point difference; LF (n.u.) = frequency-domain parameters of normalized low-frequency band; HF (n.u.) = frequency-domain parameters of normalized high-frequency band; SD1 = the standard deviation of Poincaré plot perpendicular to the line-of-identity quantifying short-term heart rate variability; SD2 = the standard deviation of the Poincaré plot along the line-of-identity quantifying long-term heart rate variability; DFA-alpha1 = an entropy parameter of detrended fluctuation analysis, measuring short-term correlations within the signal; DFA-alpha2 = non-linear entropy parameter of detrended fluctuation analysis, measuring long-term correlations within the signal; PNS index = parasympathetic nervous system index; SNS index = sympathetic nervous system index; Stress index = Baevsky’s stress index, a measure of HRV reflecting cardiovascular system stress; 2UV = a non-linear symbolic index of transformed a time series (RR intervals) into patterns with two unlike variations; * *p* ≤ 0.05 is considered significant, IQR = interquartile range.

**Table 3:** Correlation matrix of HRV and immune–inflammatory variables in bladder cancer patients, adjusted for age and sex.

	NLR	SII	PLR	LMR	PMR	NMR	sTREM-1	sTREM-1/sTREM-2	Fractalkine
Mean HR	0.22	0.27 *	0.19	−0.11	−0.06	−0.06	0.31 *	0.32 *	0.31 *
RMMSD	−0.27 *	−0.26	−0.16	−0.14	−0.12	−0.11	−0.16	−0.17	−0.14
ACmod	0.31 *	0.29 *	0.18	−0.16	−0.15	−0.13	−0.16	−0.18	−0.14
DCmod	−0.27 *	−0.26	−0.17	−0.14	−0.11	−0.1	−0.16	−0.18	−0.14
LF	0.33 *	0.3 *	0.41 *	−0.3 *	−0.21	−0.26	−0.01	−0.02	−0.02
HF	−0.33 *	−0.3 *	−0.41 *	0.3 *	0.21	−0.26	−0.01	−0.02	−0.02
SD1	−0.27 *	−0.26	−0.16	−0.14	−0.12	−0.1	−0.16	−0.17	−0.14
PNS index	−0.26	−0.27 *	−0.19	0.16	−0.12	−0.11	−0.2	−0.21	−0.19
SNS index	0.22	0.25	−0.14	−0.09	−0.04	−0.04	0.52 *	0.53 *	0.51 *
Stress index	0.19	0.21	−0.09	−0.05	−0.01	−0.01	0.54 *	0.56 *	0.53 *
2UV%	−0.01	−0.02	−0.07	0.38 *	0.34 *	0.36 *	−0.01	−0.01	−0.01

Note: sTREM-1,2 = soluble triggering receptor expressed on myeloid cells 1 and 2; fractalkine = fractalkine/CX3CL1; NLR = neutrophil-to-lymphocyte ratio; SII = systemic immune-inflammation index; PLR = platelet-to-lymphocyte ratio; LMR = lymphocyte-to-monocyte ratio; PMR = platelet-to-monocyte ratio; NMR = neutrophil-to-monocyte ratio; Mean HR = mean heart rate; RMSSD = root mean square of successive differences between normal heartbeats; ACmod = modified acceleration capacity (AC) computed as a two-point difference; DCmod = modified deceleration capacity (DC) computed as a two-point difference; LF, HF = low and high frequency band powers in normalized units; LF/HF = low to high power ratio; PNS index = parasympathetic nervous system index; SNS index = sympathetic nervous system index; Stress index = Baevsky’s stress index, a measure of HRV reflecting cardiovascular system stress; 2UV% = a non-linear symbolic index of transformed a time series (RR intervals) into patterns with two unlike variations; Pearson correlation coefficient, r; * *p* ≤ 0.05 is considered significant. Blue background color = positive correlation; Orange background color = negative correlation.

**Table 4:** Correlation matrix of psychological variables in bladder cancer patients, adjusted for age and sex.

	Physical Function T Score	Anxiety T Score	Depression T Score	Fatique T Score	Social Participation T Score	Friends’ Social Support
Mean HR	−0.14	−0.39 *	−0.29 *	−0.26	−0.28 *	−0.42 *
SDNN	0.09	0.27 *	0.22	0.18	0.05	0.11
RMMSD	0.08	0.34 *	0.25	0.11	0.06	0.11
ACmod	−0.09	−0.34 *	−0.24	−0.11	−0.05	−0.12
DCmod	0.07	0.34 *	0.25	0.12	0.04	0.08
LF	−0.08	−0.27 *	−0.24	−0.03	−0.10	−0.21
HF	0.08	0.27 *	0.24	0.03	0.10	0.21
LF/HF	0.05	−0.38 *	−0.29 *	0.01	−0.07	−0.12
SD1	0.09	0.33 *	0.25	0.11	0.06	0.11
0V%	−0.05	−0.38 *	−0.35 *	−0.11	−0.16	−0.13
2UV%	0.09	0.30 *	0.26	−0.03	0.20	0.14
Sample entropy	0.07	0.36 *	0.29 *	0.08	0.15	0.01
PNS index	0.14	0.36 *	0.27 *	0.16	0.20	0.34 *
SNS index	−0.15	−0.34 *	−0.29 *	−0.30 *	−0.25	−0.36 *
Stress index	−0.14	−0.26	−0.24	−0.28 *	−0.19	−0.28 *
sTREM-1	−0.24	0.01	0.03	−0.30 *	−0.22	−0.36 *
sTREM-1/sTREM-2	−0.30 *	0.00	0.00	−0.32 *	−0.23	−0.40 *
Fractalkine	−0.22	0.01	0.02	−0.29 *	−0.23	−0.36 *
BDNF	0.27 *	0.04	−0.05	0.08	0.18	0.19
SII	−0.19	−0.05	0.07	−0.05	−0.04	−0.27 *

Note: sTREM-1,2 = soluble triggering receptor expressed on myeloid cells 1 and 2; fractalkine = fractalkine/CX3CL1; BDNF = brain-derived neurotrophic factor; SII = systemic immune-inflammation index; Mean HR = mean heart rate; SDNN—the standard deviation of NN intervals; RMSSD = root mean square of successive differences between normal heartbeats; ACmod = modified acceleration capacity (AC) computed as a two-point difference; DCmod = modified deceleration capacity (DC) computed as a two-point difference; LF, HF = low and high frequency band powers in normalized units; LF/HF = low to high power ratio; SD1 = the standard deviation of Poincaré plot perpendicular to the line-of-identity quantifying short-term heart rate variability; 0V% = a non-linear symbolic index of transformed a time series (RR intervals) into patterns with no variations; 2UV% = a non-linear symbolic index of transformed a time series (RR intervals) into patterns with two unlike variations; Sample entropy = a non-linear HRV parameter, measuring the regularity and complexity of a time series; PNS index = parasympathetic nervous system index; SNS index = sympathetic nervous system index; Stress index = Baevsky’s stress index, a measure of HRV reflecting cardiovascular system stress;; Pearson correlation coefficient, r; * *p* ≤ 0.05 is considered significant. Blue background color = positive correlation; Orange background color = negative correlation.

## Data Availability

The data that supports the findings of this study are available within the article. The raw data of this study are available on request from the corresponding author due to privacy restrictions; minimal dataset was provided.
